# Dietary oleic acid intake increases the proportion of type 1 and 2X muscle fibers in mice

**DOI:** 10.1038/s41598-023-50464-y

**Published:** 2024-01-08

**Authors:** Yusuke Komiya, Shugo Iseki, Masaru Ochiai, Yume Takahashi, Issei Yokoyama, Takahiro Suzuki, Ryuichi Tatsumi, Shoko Sawano, Wataru Mizunoya, Keizo Arihara

**Affiliations:** 1 Laboratory of Food Function and Safety, Department of Animal Science, School of Veterinary Medicine, Kitasato University, Towada, Japan; 2 Laboratory of Animal and Human Nutritional Physiology, Department of Animal Science, School of Veterinary Medicine, Kitasato University, Towada, Japan; 3 Laboratory of Muscle and Meat Science, Department of Animal and Marine Bioresource Sciences, Faculty of Agriculture, Graduate School of Agriculture, Kyushu University, Fukuoka, Japan; 4 Laboratory of Food Health Science, Department of Food and Life Science, School of Life and Environmental Science, Azabu University, Sagamihara, Japan; 5 Laboratory of Food Science, Department of Animal Science and Biotechnology, School of Veterinary Medicine, Azabu University, Sagamihara, Japan

**Keywords:** Nutrition, Metabolism

## Abstract

Skeletal muscle is one of the largest metabolic tissues in mammals and is composed of four different types of muscle fibers (types 1, 2A, 2X, and 2B); however, type 2B is absent in humans. Given that slow-twitch fibers are superior to fast-twitch fibers in terms of oxidative metabolism and are rich in mitochondria, shift of muscle fiber types in direction towards slower fiber types improves metabolic disorders and endurance capacity. We previously had reported that oleic acid supplementation increases type 1 fiber formation in C2C12 myotubes; however, its function still remains unclear. This study aimed to determine the effect of oleic acid on the muscle fiber types and endurance capacity. An *in vivo* mouse model was used, and mice were fed a 10% oleic acid diet for 4 weeks. Two different skeletal muscles, slow soleus muscle with the predominance of slow-twitch fibers and fast extensor digitorum longus (EDL) muscle with the predominance of fast-twitch fibers, were used. We found that dietary oleic acid intake improved running endurance and altered fiber type composition of muscles, the proportion of type 1 and 2X fibers increased in the soleus muscle and type 2X increased in the EDL muscle. The fiber type shift in the EDL muscle was accompanied by an increased muscle TAG content. In addition, blood triacylglycerol (TAG) and non-esterified fatty acid levels decreased during exercise. These changes suggested that lipid utilization as an energy substrate was enhanced by oleic acid. Increased proliferator-activated receptor γ coactivator-1β protein levels were observed in the EDL muscle, which potentially enhanced the fiber type transitions towards type 2X and muscle TAG content. In conclusion, dietary oleic acid intake improved running endurance with the changes of muscle fiber type shares in mice. This study elucidated a novel functionality of oleic acid in skeletal muscle fiber types. Further studies are required to elucidate the underlying mechanisms. Our findings have the potential to contribute to the field of health and sports science through nutritional approaches, such as the development of supplements aimed at improving muscle function.

## Introduction

Chronic physical inactivity, such as long-term desk-based work, induces perturbations in skeletal muscle metabolism. These disruptions include decreased energy expenditure, an imbalance between protein synthesis and degradation, and reduced mitochondrial density and function^1^. Impaired muscle metabolism has a negative impact on the quality of life, and increases the risk of various metabolic diseases, such as type-2 diabetes and cardiovascular diseases^2^, which are serious public health and economic concerns. Improving muscle metabolism can partially rectify common metabolic diseases^2–4^, and we believe that improvement of muscle metabolism is the key to solving these problems. Contractile function and high energy consumption are two important skeletal muscle characteristics. High energy consumption is achieved through muscle contractions during physical activity and posture maintenance, as well as through heat production as part of basal metabolism, which primarily utilizes oxidative phosphorylation and glycolysis. These metabolic characteristics and contraction rates depend on the fiber type composition of skeletal muscles^5^. In mammalian skeletal muscles, fibers are classified into two main types, namely types 1 and 2. Type 1 fibers are rich in mitochondria, have a high oxidative capacity, and are resistant to fatigue. Type 2 fibers are further subdivided into types 2A, 2X, and 2B^5^. Type 2B fibers exhibit high glycolytic metabolism and fatigue, and fastest twitch. In terms of contractile characteristics, types 2A and 2X fibers have intermediate characteristics between those of types 1 and 2B fibers; therefore, fast-twitch fibers increase in the order of 2A < 2X < 2B. Although type 2X fibers are superior to glycolysis and type 2A fibers are oriented toward oxidative metabolism, the metabolic characteristics do not correlate perfectly with the contractile characteristics. Rat muscles with predominantly more type 2A fibers are more oxidative (based on citrate synthase activity) than muscles with predominantly more type 1 fibers^6^. In human muscles, type 2B fibers are not detectable, although the corresponding myosin heavy chain (MyHC) 2B gene is present in the genome, and fibers typed as 2B based on myosin ATPase staining are in fact 2X fibers based on MyHC composition^7,8^.

These fiber types are generally classified according to their MyHC isoforms. To date, four adult MyHC isoforms have been identified in rodent skeletal muscles, namely MyHC1, 2A, 2X, and 2B, which respectively determine fiber type 1, 2A, 2X, and 2B. The composition of a muscle in terms of the proportions of fiber types determines the various contractile and metabolic properties of skeletal muscles, including high-speed muscle strength and endurance performance. An increase in type 1 fibers contributes to improved endurance performance, fatigue resistance, and oxidative metabolism^9^.

Exercise improves muscle properties, such as contraction, metabolism, and function^10^. For example, resistance training increases muscle fiber size and maximal tension output via stimulation of muscle protein synthesis^11,12^. Moreover, endurance training increases mitochondria content, leading to improvements in oxidative metabolism and endurance capacity^10^. Notably, endurance training markedly increases the oxidative potential of type 2X and 2B fibers, resulting in an oxidation potential that markedly surpasses the aerobic capacity of type 1 fibers of untrained individuals^13^. Therefore, in humans, the activities of both oxidative and glycolytic enzymes in all muscle fiber types is sufficiently large to accommodate a substantial range of aerobic and anaerobic metabolism. In addition to exercise, various dietary components can alter muscle characteristics; such foods are known as exercise mimetics. For example, the flavonoid (–)-epicatechin improved mitochondrial function and increased capillary formation in skeletal muscle^14^. In addition, resveratrol (3,5,4’-trihydroxystilbene) enhances mitochondrial biogenesis, stimulates angiogenesis, improves exercise capacity, and increases insulin sensitivity in the same manner as exercise training^14,15^. Previously, we found that dietary olive oil intake improved running endurance by increasing muscle triacylglycerol (TAG) accumulation in mice^16^. Furthermore, supplementation with oleic acid, which is abundant in olive oil, improves mitochondrial maximal respiration and increases type 1 fiber formation in myotubes differentiated from C2C12 myoblasts^17^. Oleic acid is a mono-unsaturated fatty acid (n-9, 18:1) and is a functional fatty acid in skeletal muscle. For example, oleic acid activates the sirtuin 1-peroxisome proliferator-activated receptor γ coactivator (PGC)-1α transcriptional complex, promotes fatty acid oxidation^18^. We reported that oleic acid was an agonist of peroxisome proliferator-activated receptor (PPAR) δ^17^, which is a nuclear receptor. Overexpression of constitutively active, muscle-specific VP16-PPARδ induces an increase in oxidative enzyme levels, and mitochondrial biogenesis together with a fiber type shift from type 2 to 1 in mice^19^. Notably, in mice, PPARδ activation promotes running endurance by preserving serum glucose levels during exercise causing promotion of fatty acid oxidation in muscle tissues^20^. Dietary oleic acid intake is speculated to alter the fiber type composition of muscles and to improve endurance capacity in vivo; however, its function still remains unclear.

In this study, we aimed to investigate the effect of oleic acid on skeletal muscles using an *in vivo* mouse model. The study focused on running performance as well as muscle fiber types and metabolism-related genes in the soleus (slow-type dominant) and extensor digitorum longus (EDL, fast-type dominant) muscle tissues. Additionally, we analyzed changes in circulating energy substrates during exercise.

## Results

### Effect of dietary oleic acid on treadmill endurance capacity and serum substrate concentrations

**Figure 1:**
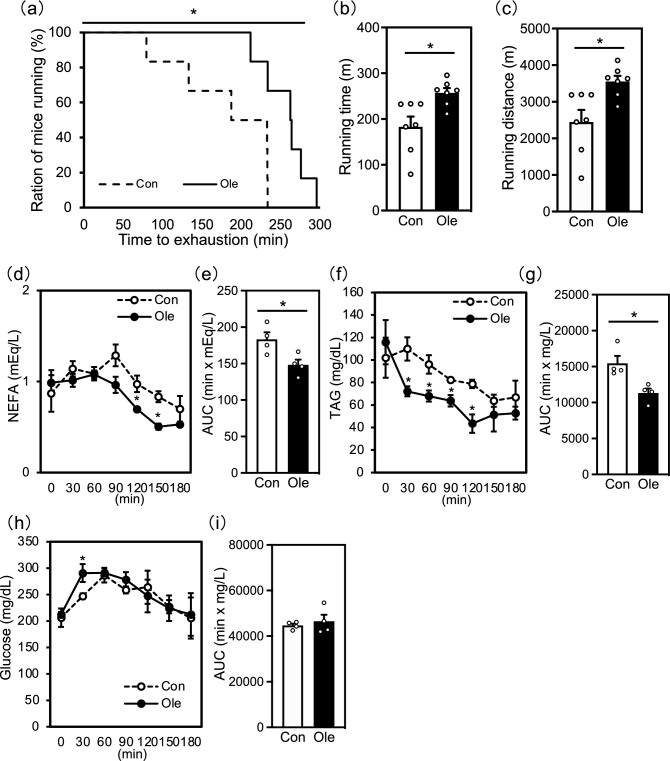
Effect of dietary oleic acid on treadmill endurance and serum substrate concentrations. (**a**) Percentage of running population on the treadmill. (**b**) Time and (**c**) distance covered until attaining exhaustion under a forced running exercise-to-exhaustion test. (**d**) Blood non-esterified fatty acid (NEFA), (**f**) triacylglycerol (TAG), and (**h**) glucose monitored during the running test in mice. The area under the curves of (**e**) NEFA, (**g**) TAG, and (**i**) glucose were determined using the trapezoidal rule. Results are presented as means ± SE (n=4–7), *p < 0.05 versus control group.

First, we examined the effects of dietary oleic acid intake on mouse endurance using a treadmill. A significant increase in the achieved running distance of mice (Fig. 1a). Moreover, the oleic acid group ran for a longer time (approximately 40%) and a farther distance (approximately 45%) than the control group ran (Fig. 1b,c), revealing that dietary oleic acid intake systemically increased endurance capacity. To determine whether improved running endurance in the oleic acid group was associated with blood substrate levels, serum concentrations of non-esterified fatty acid (NEFA), TAG, and glucose were analyzed during exercise. We observed decreased blood concentrations of NEFA (at 120 and 150 min) and TAG (at 30, 60, 90, and 120 min) in oleic acid-fed mice during exercise (Fig. 1d and f). Furthermore, area under the curve (AUC) values of blood NEFA and TAG decreased in the oleic acid group (Fig. 1e,g). Blood glucose concentrations were significantly higher in the oleic acid group at 30 min that in the control group; however, the AUC value did not change between the two groups (Fig. 1h,i).

### Skeletal muscle fiber type regulation by oleic acid

**Figure 2:**
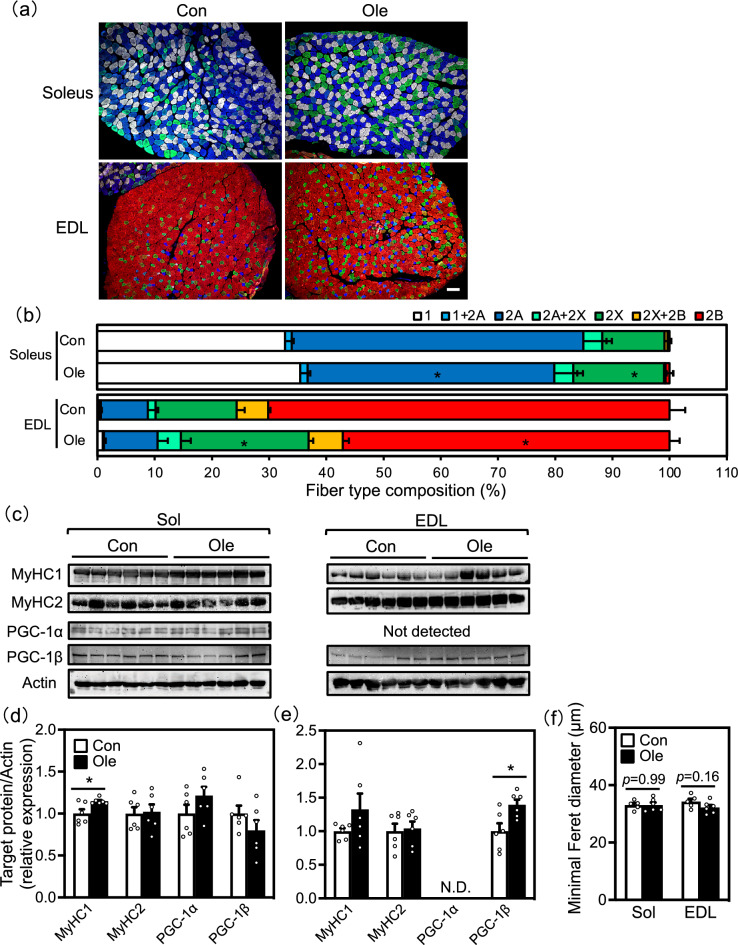
Skeletal muscle fiber type determination and the expression levels of muscle fiber type-regulators. (**a**) Myosin heavy chain (MyHC) isoform expression (MyHC1 [white], MyHC2A [blue], MyHC2X [green], and MyHC2B [red]) in soleus and extensor digitorum longus (EDL) muscle. The bars indicate 100 μm. (**b**) Fiber-type composition of soleus (upper box) and EDL muscle (lower box). (**c**) Protein expression levels of MyHC1, peroxisome proliferator-activated receptor γ coactivator (PGC)-1α, PGC-1β, and actin (loading control). (**d** and **e**) The densitometric quantification of images of panel c. (**f**) The minimal Feret diameter means of soleus and EDL muscle. Results are presented as means ± SE (n=5–6), *p<0.05 versus control group. The original imaging representing immunoblots are shown under Supplementary Information.

To determine whether dietary oleic acid-improved running endurance was associated with muscle fiber type transitions, we analysed expressionof MyHC, which is a marker of muscle fiber type^21,22^. Immunohistochemical analysisrevealed that oleic acid intake increased the number of MyHC2X-positive fibers and decreased the number of MyHC2A-positive fibers in the slow soleus muscle (Fig. 2a,b). In addition, western blot analysis showed that oleic acid intake increased MyHC1 expression levels only in the soleus muscle (Fig. 2c,d). In the fast EDL muscle, the proportion of MyHC2X increased and that of MyHC2B decreased in the oleic acid group (Fig. 2a,b). MyHC2 expression levels did not change in both muscles according to western blot analysis (Fig. 2c,d,e). To confirm whether changes in muscle fiber type were associated with an increase in muscle mass, we measured the minimal Feret diameter of muscle fibers. The results showed no significant changes in either the soleus muscle or the EDL (Fig. 2f). In addition, there were no significant differences in the ratio of muscle weight to body weight between the two groups (Table 1). This suggests that the changes in muscle fiber type are indicative of a proportional shift rather than absolute changes in muscle mass.

Considering that the oleic acid intake altered the muscle fiber type transitions or MyHC genes expression, we examined the expression levels of MyHC1 and 2X regulators to determine the underlying mechanism. Recent studies have implicated the transcriptional coactivator PGC-1α in muscle fiber-type switching and determination, especially in type 1 fibers^23^. PGC-1β was later identified by homology of PGC-1α^24,25^ and was known to drive the formation of highly oxidative fibers, specifically type 2X fibers^26^. Therefore, we analyzed the expression of these factors at the protein level. On one hand, increased PGC-1β expression was observed in the EDL muscle of the oleic acid-fed group (Fig. 2c,e). On the other hand, there was no significant difference in PGC-1α and PGC-1β expression in the soleus muscle of the two groups (Fig. 2c,e).

### Muscle TAG accumulation by oleic acid

**Figure 3:**
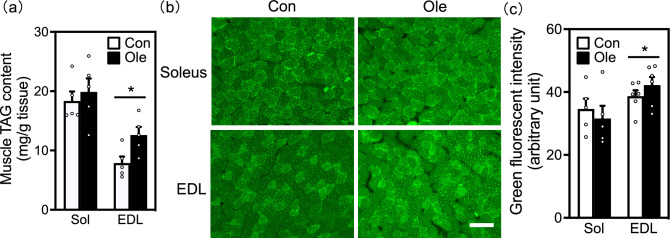
Effect of dietary oleic acid on the muscle triacylglycerol (TAG) content. (**a**) Whole TAG content in soleus and extensor digitorum longus (EDL) muscles (mg/g tissue). (**b**) Representative images of muscle specimens stained with BODIPY493/503 in mouse soleus and EDL muscles. The bar indicates 100 μm. (**c**) Quantification of muscle lipid content in muscle fibers. The green fluorescence intensity in the randomly chosen cross-sectional muscle fibers was quantified and standardized to the area of each muscle fiber (five fields/tissue). Results are presented as means ± SE (n=5–6), *p < 0.05 versus control group.

We examined the effects of dietary oleic acid intake on TAG content of the muscle. Seeing that muscle TAG pools adaptively increased in response to endurance training^27–29^, we hypothesized that increased TAG content upon oleic acid intake would contribute to improved running endurance. To test this hypothesis, we analyzed muscular TAG content using biochemical analysis and BODIPY493/503 staining of the soleus and EDL muscles. We found that oleic acid intake increased TAG content in the EDL muscle only (Fig. 3a). Green fluorescence intensity upon BODIPY 493/503 staining was significantly higher in the EDL muscle of the oleic acid group than in the control group (Fig. 3b,c). There was no significant difference in TAG content in the soleus muscles of the two groups.

### Effect of dietary oleic acid on the expression levels of metabolism-related factors

**Figure 4:**
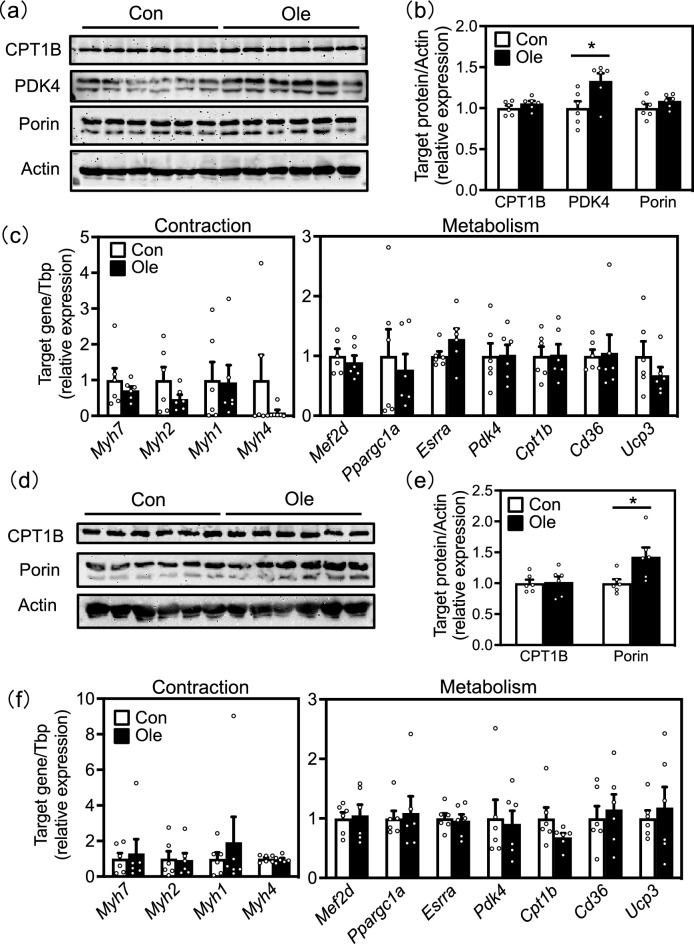
Effect of dietary oleic acid on the expression levels of metabolism-related factors. (**a**) Protein expression levels of carnitine palmitoyltransferase (CPT)1B, pyruvate dehydrogenase kinase (PDK)4, porin, and actin (loading control) in soleus muscle. (**b**) The densitometry quantification of images of panel a. (**c**) RNA expression levels of genes related to muscle contractile proteins and metabolism in the soleus muscle demonstrated by reverse transcription polymerase chain reaction (RT-PCR). (**d**) Protein expression levels of CPT1B, porin, and actin (loading control) in EDL muscle. (**e**) The densitometry quantification of images of panel d. (**f**) RNA expression levels of genes related to muscle contractile proteins and metabolism in EDL muscle demonstrated by RT-PCR. Results are presented as means ± SE (n=6), *, *p* < 0.05 vs. control group. The original imaging representing immunoblots are shown under Supplementary Information.

As skeletal muscle fiber types are closely related to muscle metabolism^5^, we analyzed the protein and mRNA expression levels of lipid metabolism-related factors using western blotting and reverse transcription-quantitative polymerase chain reaction (RT-qPCR) to understand the changes in muscle metabolism with oleic acid intake. In the soleus muscle, the protein expression levels of pyruvate dehydrogenase kinase (PDK)4 increased in the oleic acid group (Fig. 4a,b). In the EDL muscle, the protein expression levels of porin increased in the oleic acid group (Fig. 4d,e). There was no significant difference in mRNA expression levels of any genes between the two groups in either the soleus or the EDL muscle (Fig. 4c,f).

## Discussion

We demonstrated that dietary oleic acid intake for 4 weeks improved endurance capacity, accompanied by alterations in muscle fiber type proportions, such as an increase in type 1 and 2X fibers in the soleus muscle, and an increase in 2X fibers in the EDL muscle. In the EDL muscle, oleic acid intake induced the upregulation of PGC-1β expression, which probably induced fiber type transitions to type 2X and the accumulation of muscle TAG content. However, the effect of dietary oleic acid on MyHC composition, intramuscular triacylglycerol (IMTG) content, and the expression of oxidative metabolism-related genes in the soleus muscle did not align with that observed in the EDL. Thus, our study indicates a dietary oleic acid-dependent, differential regulation of contractile and metabolic properties between fast- and slow-type muscles. While responses vary by muscle site, we believe that the shift of muscle fiber types in direction towards slower fiber types and an increase in lipid utilization in skeletal muscles contributed to the improvement in running endurance.

Elucidation of the mechanisms involved in the coordinated regulation of muscle metabolic and structural programs during fiber type transitions has implications for new therapeutic approaches to many human diseases, including metabolic disorders and obesity. Muscle fiber type composition is influenced by both genetic factors and postnatal environmental factors, such as exercise^22,30^. Nuclear receptors, such as PPARs and estrogen-related receptors, along with their co-regulators, PGC-1s and adenosine monophosphate-activated protein kinase, are key regulators of muscle energy metabolism^2,31,32^. Exercising, including both endurance and resistance training, activates these factors and uniformly induces muscle fiber type transitions toward the slower type, regardless of the existing fiber type compositions^33,34^. However, interestingly, oleic acid intake increased type 2X fibers in both the soleus (slow-twitch fiber-predominant tissue) and EDL (fast-twitch fiber-predominant tissue) muscle, and increased PGC-1β expression, especially in the EDL muscle. Arany et al. reported that tibialis anterior muscles of the transgenic expression of PGC-1β mice showed a remarkable increase in the proportion of type 2X fibers, which are oxidative, but have fast-twitch biophysical properties and improved running endurance^26^. In addition, activation of the MyHC2X promoter was almost entirely due to the coactivation of myocyte enhancer factor 2D (MEF2D) by PGC-1β^26^. We had previously reported that oleic acid supplementation increases MEF2D expression in C2C12 myoblasts^17^. On one hand, the reports suggested that oleic acid intake may increase the proportion of type 2X fibers via the activation of PGC-1β and MEF2D. On the other hand, overexpression of PGC-1β negatively regulates MyHC2x mRNA expression in L6 myoblasts^35^. The effect of PGC-1β on muscle fiber types has not been further reported and there is no conclusive evidence of its function in muscle fiber types. The main difference in the reports regarding the regulation of muscle fiber types by PGC-1β was that Mortensen et al. conducted their experiments in vitro, whereas Arany et al. conducted experiments in vivo. Therefore, to positively regulate the contractile protein MyHC2X and increase PGC-1β expression, it may be necessary to consider other factors such as neural control and physical stimuli that were not present in the in vitro experiments. However, increased PGC-1β is associated with oxidative metabolism in skeletal muscle, as evidenced by its ability to increase citric acid synthase activity in L6 cells^35^ and to increase mRNA expression related to oxidative phosphorylation enzymes and lipid oxidation in the quadriceps muscle^26^.

Another important factor is PPARδ, and oleic acid is a ligand for PPARδ^17^. Muscle-specific overexpression of PPARδ in mice leads to a shift of muscle fiber types in direction towards slower fiber types^19^; however, administration of a PPARδ agonist (GW1516) alone does not affect the proportion of type 1 fibers in mice^36^. Similarly, we have demonstrated that PPARδ is not involved in the oleic acid-induced increase of MyHC1 in C2C12 cells according to RNA interference experiments^17^. On the contrary, Wang et al. observed an increase in type1 fibers (troponin I) with oral administration of the PPARδ-specific agonist GW501516^19^, and Chen et al. reported a similar increase in type I fibers using SDH staining with GW501516 administration alone^37^. These findings highlight the complexity of the relationship between the activation of PPARδ by agonists such as drugs or fatty acids and the regulation of muscle fiber types. Further research is therefore needed to fully understand how PPARδ influences the regulation of muscle fiber types. Furthermore, PPARδ has been shown to induce the mRNA expression of factors related to lipid metabolism, such as Pdk4 and malonyl-CoA decarboxylase, in a ligand-dependent manner^20^. Given that the oleic acid-induced increase in expression of factors related to lipid metabolism, including PDK4, is mediated through PPARδ activation^17^, it is plausible to conclude that the increased PDK4 protein expression observed in this experiment is also a result of PPARδ activation. In addition, PPARδ causes the sparing of blood glucose and promotes endurance capacity through a shift in muscle fiber characteristics toward oxidative fibers and an increase in the oxidative capacity of skeletal muscle in mice^20^. We also measured the concentration of circulating substrates during exercise, but contrary to our expectations, there was no significant change in glucose concentration. Instead, we observed a decrease in TAG and NEFA concentrations. Romijn et al. demonstrated that plasma NEFA is most important during aerobic exercise^38^, and there was a gradual increase in the reliance on plasma free fatty acids and glucose as energy substrates during exercise at 65% maximum rate of oxygen consumption (VO_2_ max) compared with 25% VO_2_ max. Interestingly, during exercise at 85% VO_2_ max, blood NEFA concentrations were shown to decrease, in contrast to the gradual increase observed at 25% and 65% VO_2_ max. The movement of blood NEFA during exercise suggests that mice receiving oleic acid may have an increased VO_2_ max during exercise. However, it's important to note that the findings reported by Romijn et al. are derived from human observations. Therefore, the direct application of these findings to the results obtained in this mouse study requires careful consideration. To validate our hypothesis that decreased blood NEFA is associated with increased VO_2_ max during exercise, it would have been desirable to measure the respiratory exchange ratio during exercise. Unfortunately, we were unable to perform this measurement, and this is one of the limitations of our study.

The characteristics of endurance athletes’ skeletal muscles, not only a high oxidative capacity but high muscle TAG accumulation as well; this is known as the athlete’s paradox^28,39^. During exercise the skeletal muscle is continuously supplied with energy derived from three main substrates: creatine phosphate, glycogen, and TAG. Fat oxidation is emerging as the second prominent substrate for endurance exercise after intramuscular glycogen. Fat, the most abundant endogenous energy store, exceeds glycogen storage by more than 60-fold in humans^40^. During endurance exercise, fatty acid oxidation in mitochondria allows for prolonged activity and delays the onset of glycogen depletion and hypoglycemia^41^. While various regulatory mechanisms elucidate the intricate interplay between carbohydrate and fat metabolism^42^, there is no doubt that muscle TAG pools serve as a crucial energy source during prolonged exercise. Several studies reported that type 1 fibers contain more muscle TAG than type 2 fibers in human^43–45^. In contrast, muscle TAG accumulation occurs mainly in type 2A and 2X fibers and intramuscular TAG content depends on the muscle fiber type composition in the soleus and EDL muscles of mice^46^. Therefore, increased TAG content was induced in the EDL muscle (change between 2B and 2X fibers), but not in the soleus muscle (change between 2A and 2X fibers), via muscle fiber type transitions. Sterol-responsive element binding protein (SREBP) is a master regulator of lipid metabolism in skeletal muscle^47,48^, and PGC-1β promotes the expression of lipid synthesis-related factors, such as enzymes of fatty acid, triglyceride synthesis, and cholesterol biosynthesis, through SREBP activation^49^. In addition, oleic acid regulates the Plin2, 4, and 5 genes via PPARδ activation in differentiated C2C12 cells^50^. These genes, especially Plin 2 and 5, are involved in lipid droplet formation in muscle cells^51–54^. Plin2 facilitates muscle lipid storage by stabilization of lipid droplets and inhibition of lipolysis^55^. Plin5, also known as a lipid droplet coat protein, has diverse functions including lipid droplet formation; however, its mechanism has not fully elucidated^55^. In the present study, oleic acid probably increased the muscle TAG content via these pathways, with an increase in type 2X fibers proportion. Furthermore, it is possible that oleic acid not only contributes to fat accumulation in skeletal muscle, but also promotes lipid utilization by increasing PDK4 expression. PDK4 inactivates the enzyme pyruvate dehydrogenase complex, which converts pyruvate from glycolysis to acetyl-CoA^56^, by phosphorylation. This action suppresses energy production from glucose and promotes lipid utilization.

The limitation of this study was that the mechanism of the shift of muscle fiber types following dietary oleic acid intake was not determined. We focused on PGC-1β expression as a factor influencing type 2X fiber induction, and we found that oleic acid intake increased PGC-1β expression only in the EDL muscle, but not in the soleus muscle. Differences in the expression levels and activity of nuclear receptors, signaling factors, and proteins related to metabolism between fiber types are occasionally observed^57–61^, along with differences in reactivity to drugs or functional foods, in tissues that predominantly have either fast- or slow-twitch fibers^62,63^. These differences in characteristics between muscle fiber types may have influenced the differences in PGC-1β expression between the slow soleus and fast EDL muscles. Furthermore, we did not determine the mechanism underlying the increased type 1 fibers in the soleus muscle. We focused on PGC-1α as an inducer of type 1 muscle fibers because muscle-specific overexpression of PGC-1α in mice leads to a shift of muscle fiber types in direction towards slower fiber types^23^. However, we did not detect a difference in PGC-1α expression associated with oleic acid intake. We have reported that oleic acid possesses ligand activity for PPARδ, and is known as a type 1 fiber inducer^19^, and have also demonstrated that PPARδ is not essential for the increase in type 1 fiber formation in C2C12 myoblasts^17^. Therefore, it is possible that changes in metabolic characteristics, such as PPARδ activation and the promotion of lipid utilization by oleic acid, occurred first, and the subsequent increase in the proportion of type 1 muscle fibers was induced as a result, rather than activation of specific factors. Furthermore, the change in muscle fiber type to slow-twitch fibers would be expected to induce an increase in mitochondria. To investigate whether the observed changes in fiber type induced by oleic acid are related to mitochondrial quantity, we examined the expression of porin, a mitochondrial membrane protein. We found a significant increase of porin expression in the EDL muscle, suggesting a potential increase in mitochondria. However, porin is not a specific marker for mitochondria as it is also expressed in other subcellular fractions (sarcoplasmic reticulum and caveolae on the sarcolemmal membrane)^64,65^. While the possibility of increased mitochondria by oleic acid intake was suggested, further investigation, including factors such as, cardiolipin or citrate synthase activity^66^, is required to definitively prove the increase in mitochondria. Another study limitation is the variability of the results due to differences in analytical methods. The increase in MyHC1 composition in the soleus muscle was confirmed by western blotting, but similar results were not obtained by immunohistochemical staining using sections. The discrepancy may be due to the difference between western blotting, which analyzes the entire muscle tissue homogenate, and staining experiments, which analyze a portion of tissue sections.

In summary, we revealed that dietary oleic acid intake improves running endurance by inducing fiber type transition, most likely induced by PGC-1β expression. This enhancement of endurance capacity is probably associated with the reduction of circulating lipids during exercise and the promotion of fat utilization in skeletal muscle, particularly through PDK4. Collectively, these findings suggest the potential of oleic acid as a novel exercise-mimetic food and its potential application as a novel approach to treating metabolic disorders and their associated complications via skeletal muscle metabolism. However, further studies are required to clarify the detailed mechanisms underlying the effects of oleic acid on skeletal muscle.

## Materials and methods

### Study design and diets

**Figure 5:**
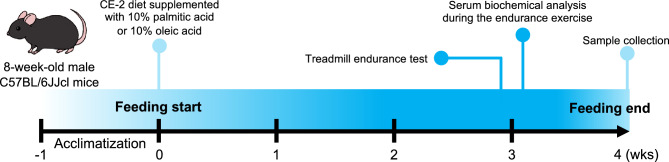
Schematic representation of experimental design. Eight-week-old male C57BL/6JJcl mice were fed a CE-2 diet supplemented with 10% (w/w) palmitic acid (control diet) or oleic acid for 4 weeks. On the last day of week3 and the first day of week 4, running endurance tests and serum biochemical analyses were performed, respectively. After a 4-week feeding period, the mice were euthanized and samples were collected.

All animal experiments were conducted in strict accordance with the Guidelines for Proper Conduct of Animal Experiments published by the Science Council of Japan and with the approval of the Animal Care and Use Committee of Kitasato University (approval no. 20-007). And all methods were performed in accordance with guidelines and regulations at Kitasato University, as well as complying with ARRIVE guidelines^67^ for the reporting of animal experiments. Eight-week-old male C57BL/6JJcl mice were purchased from CLEA Japan, Inc. (Tokyo, Japan). The experimental design is illustrated in Fig. 5. The mice were housed in plastic cages in an animal room at 22 ± 2 °C and 50 ± 10% humidity under an artificial lighting system of 12-h light/12-h dark cycle. They were acclimated to the environment for one week. Following the acclimatization period, the mice were fed a CE-2 (CLEA Japan, Inc.) diet supplemented with 10% (w/w) palmitic acid (control diet) or oleic acid for 4 weeks. Palmitic acid was chosen as control fatty acid because it is a representative saturated fatty acid commonly found in soybean oil and olive oil, which has been used in previous studies^16^. Although we considered using linoleic acid as a control, we decided against it because unsaturated fatty acids have ligand activity for PPARs^68^. In our previous study, we reported the results of an 8-week feeding test^16^, and in a preliminary study we confirmed similar changes in skeletal muscle characteristics even with a 4-week period. Therefore, we chose a shorter period of 4 weeks for this study. Nutritional and fatty acid compositions of experimental diets are shown in Table 2 and 3, respectively. Running endurance tests and serum biochemical analyses were performed on the last day of week 3 and the first day of week 4, respectively. After a 4-week feeding period, the mice were euthanized by cervical dislocation under inhalation anesthesia with isoflurane. The adipose tissues, liver, and skeletal muscles (soleus, EDL, and gastrocnemius muscles) were harvested and weighed immediately. The growth performance and tissue weight are shown in Table 1. All tissues were stored at –80 °C until further analysis.

### Treadmill endurance test

The treadmill endurance test was performed as described previously^16^. Mice were acclimated to treadmill running (10 m/min for 10 min) two days before the test. For the endurance test, the mice ran on a treadmill at a 5° incline, and the speed was gradually increased from 10 to 15 m/min. After reaching a speed of 15 m/min, the mice were made to run at a constant speed till exhaustion. When a mouse was unable to avoid electrical stimulation for more than 5 s, it was considered to be in a state of exhaustion. The electrode that accompanied by the treadmill (model MK-690S; Muromachi Kikai Co., Ltd., Tokyo, Japan) detected instances in which avoidance exceeded 5 s, ensuring the elimination of human bias. Endurance was measured as a function of time and distance.

### Serum biochemical analysis

Serum biochemical components were monitored during the endurance exercise (10–15 m/min for 180 min). Blood was collected from the tail at 0, 30, 60, 90, 120, 150, and 180 min after starting the exercise, and the levels of blood glucose, TAG, and NEFA were measured using commercial kits (LabAssay^TM^ Glucose: 298-65701, LabAssay^TM^ Triglyceride: 290-63701, and LabAssay^TM^ NEFA: 294-63601, respectively; FUJIFILM Wako Pure Chemical Corporation, Osaka, Japan).

### RNA isolation and RT-qPCR

The analysis of RNA expression levels of specific genes, presented in Table 4, was performed as described previously^16^. EDL and soleus muscle tissues were pulverized under liquid nitrogen, then total RNA was extracted from them using the ISOGENII kit (NIPPON GENE, Tokyo, Japan) and reverse transcribed using SuperScript III Reverse Transcriptase (Invitrogen, Grand Island, NY, USA) and Oligo d(T)12–18 primers (Applied Biosystems, Waltham, MA, USA), according to the manufacturer’s instructions. RT-qPCR was performed using an Applied Biosystems StepOnePlus system with PowerUp SYBR Green Master Mix (Thermo Fisher Scientific, Waltham, MA, USA). All primers were designed using ProbeFinder software (version 2.53; Roche Diagnostics GmbH, Mannheim, Germany) and an intron-spanning assay. The primer sets used in this study are listed in Table 4. Amplicon specificity was verified using melting curve analysis. Genes were analyzed using a standard curve constructed from a serial dilution of complementary DNA aliquots pooled from one randomly chosen sample. TATA box-binding protein was used as an internal standard.

### Western blotting

EDL and soleus muscle tissues were pulverized under liquid nitrogen, and crushed muscles (approximately 50 mg) were homogenized in sodium dodecyl sulfate (SDS) solution containing 10% SDS, 40 mM dithiothreitol, 5 mM ethylenediaminetetraacetic acid, and 0.1 M Tris-HCl buffer (pH 8.0), in which a protease inhibitor cocktail (for use with mammalian cell and tissue extracts; Nacalai Tesque, Inc., Kyoto, Japan) was added at 1:100. The homogenates were heated in boiling water for 3 min. The prepared protein samples (8 μg/μL) were separated via 10% SDS-polyacrylamide gel electrophoresis under reducing conditions and transferred onto polyvinylidene fluoride membranes (Bio-Rad, Hercules, CA, USA). The membranes were then incubated with a blocking reagent (5% powdered skim milk in tween tris buffered saline) for 45 min before incubation with primary antibodies diluted in Can Get Signal solution 1 (Toyobo, Osaka, Japan) overnight at 4 °C. The following antibodies were used: mouse monoclonal anti-actin (1:10000 dilution; Chemicon MAB1501, [clone C4]), anti-porin (1:2000 dilution; Abcam ab14734, Cambridge, UK), anti-MyHC1 (1:5000; Sigma-Aldrich M8421, [clone NOQ7.5.4D], St. Louis, MO, USA), anti-MyHC2 (1:5000 dilution; Sigma-Aldrich M4276, [clone MY32]), anti-PGC1α (1:5000 dilution; Proteintech 66369-1-Ig, Rosemont, IL, USA), anti-PGC1β (1:5000 dilution; Proteintech 67821-1-Ig), rabbit polyclonal anti-PDK4 (1:2000 dilution; Proteintech, 12949-1-AP), and anti-carnitine palmitoyltransferase 1B (1:5000 dilution; Proteintech 22170-1-AP). The membranes were then incubated for 1 h with peroxidase-conjugated anti-mouse IgG (1:5000 dilution; Jackson ImmunoResearch 287695, West Grove, PA, USA) or anti-rabbit IgG secondary antibody (1:2000 dilution; Dako, Santa Clara, CA, USA) diluted in Can Get Signal Solution 2 (Toyobo). The bands were detected using enhanced chemiluminescence (GE Healthcare, Chicago, IL, USA). Intensity of the bands was quantified using the ImageJ software (National Institutes of Health, Bethesda, MD, USA) and normalized with respect to actin as a loading control. Contrary to our expectations, two bands were detected for PDK4 and PGC1α. However, it was difficult to identify them based on band size alone; thus, both bands were analyzed.

### Muscle TAG content

Total lipids were extracted using the Folch method with minor modifications^16^. All connective tissues, fat, and nonmuscle tissues were removed from EDL and soleus muscle tissues using micro scissors. Cleaned muscle tissues were pulverized under liquid nitrogen, and crushed muscle tissues (approximately 50 mg) were homogenized in 1.5 mL of chloroform/methanol (2/1, v/v), and incubated overnight at 4 °C. Thereafter, 250 μL of 0.9% NaCl solution was added to the incubated chloroform/methanol solutions and mixed by vortexing. After centrifugation, 600 μL of the lower phase containing neutral lipids was dried using a depression aspirator (A-3S; Tokyo Rikakikai Co. Ltd., Tokyo, Japan). To determine the TAG content, the lipid pellet was solubilized in Triton X-100, and TAG concentrations were determined using a commercial kit (Fujifilm Wako Pure Chemicals Ltd.) as per the manufacturer’s instructions. Muscle TAG content was normalized to the wet muscle tissue weight.

### Preparation of muscle sections and BODIPY 493/503 staining

The harvested EDL and soleus muscles were embedded in Tissue-Tek O.C.T Compound (Sakura Finetek Japan, Tokyo, Japan), frozen in isopentane, and cooled with liquid nitrogen. Cryosections from each block were collected on Matsunami adhesive silane -coated glass slides (S9226; Matsunami Glass Ind., Ltd., Osaka, Japan). The staining of IMTG was performed as described previously^16^. Muscle sections were fixed with 4% paraformaldehyde for 20 min at 4 °C. The plates were incubated with BODIPY 493/503 (Life Technologies, Carlsbad, CA, USA) for 90 min. All samples were mounted in ProLong^TM^ Diamond antifade reagent with DAPI (Invitrogen). Stained IMTG was visualized using a KEYENCE BZ-X810 fluorescence microscope (KEYENCE, Osaka, Japan). Calculations of IMTG content were performed using ImageJ 1.45f software (Rasband W; National Institutes of Health), by densitometry of BODIPY 493/503 fluorescence intensity, and standardized to the area.

### Muscle fiber-type determination by immunohistochemistry

Four MyHC isoforms were determined by “stained glass-like staining,” as reported by Sawano et al.^69^, with minor modifications. Briefly, the EDL and soleus muscle sections were fixed with steam for 5 min, and the slides were incubated in 1.0% Triton X-100 in phosphate-buffered saline (PBS) at room temperature for 10 min. Subsequently, the slides were incubated with blocking solution (3% bovine serum albumin [BSA], 0.1% Triton X-100, and 0.05% Tween 20 in PBS; pH 7.2) for 1 h at room temperature. The slides were then incubated overnight at 4 ℃ in a mixture of home-made primary monoclonal antibodies against MyHC 1 , 2A, 2X, and 2B that were prelabeled with Molecular Probes Alexa Fluor 647, Alexa Fluor 350, Fluorescein 500, and HyLyte Fluor 594, respectively (1:100 dilution in sterile solution of 1% BSA, 0.1% cold fish skin gelatin, 0.5% Triton X-100, 0.05% Tween 20, 0.01% biotin, and 0.05% sodium azide in PBS). Sections were mounted in ProLong Diamond Antifade Reagent (Invitrogen) and observed under a KEYENCE BZ-X810 fluorescence microscope. Slow fibers (type 1) and fast fibers (types 2A, 2X, and 2B) on the whole muscle cross sectional area were eventually counted and the counting was blinded to the mouse diets. Minimal Feret diameter of muscles were measured using ImageJ 1.45f software.

### Statistics

Data are expressed as mean ± standard error (SE). Experimental results were analyzed using Student’s *t*-test. Statistical significance was set at *p* < 0.05, and statistically significant differences with *p* < 0.05 are indicated by an asterisk. All statistical analyses were performed using the Excel‐Toukei ver.7.0 software (Social Survey Research Information Co. Ltd., Tokyo, Japan).

### Supplementary Information

Supplementary Information.

## Data Availability

All relevant data generated or analyzed during this study are included in this published article and its Supplementary Information file.
