# Supplementary material for: Dietary oleic acid intake increases the proportion of type 1 and 2X muscle fibers in mice
Source: Sci Rep. 2024 Jan 8;14:755. doi: 10.1038/s41598-023-50464-y (PMC10774392; doi:10.1038/s41598-023-50464-y)
Supplement: Supplementary file 1 — Supplementary Information. [file 41598_2023_50464_MOESM1_ESM.docx]

**Supplementary Information**

**Dietary oleic acid intake increases the proportion of type 1 and 2X muscle fibers in mice**

Yusuke Komiya^1^*, Shugo Iseki^1^, Masaru Ochiai^2^, Yume Takahashi^1^, Issei Yokoyama^1^, Takahiro Suzuki^3^, Ryuichi Tatsumi^3^, Shoko Sawano^4^, Wataru Mizunoya^5^, Keizo Arihara^1^

^1^Laboratory of Food Function and Safety, Department of Animal Science, School of Veterinary Medicine, Kitasato University, Towada, Japan

^2^Laboratory of Animal and Human Nutritional Physiology, Department of Animal Science, School of Veterinary Medicine, Kitasato University, Towada, Japan

^3^Laboratory of Muscle and Meat Science, Department of Animal and Marine Bioresource Sciences, Faculty of Agriculture, Graduate School of Agriculture, Kyushu University, Fukuoka, Japan

^4^Laboratory of Food Health Science, Department of Food and Life Science, School of Life and Environmental Science, Azabu University, Sagamihara, Japan

^5^Laboratory of Food Science, Department of Animal Science and Biotechnology, School of Veterinary Medicine, Azabu University, Sagamihara, Japan

***Corresponding author:**

Yusuke Komiya

Department of Animal Science, School of Veterinary Medicine, Kitasato University, Towada 034-8628, Japan, Telephone number: +81-176-23-9341, Fax number: +81-176-23-8703, E-mail address: komiya@vmas.kitasato-u.ac.jp

**Western blotting raw data**

**＜Fig. 2C Soleus MyHC1＞**

**
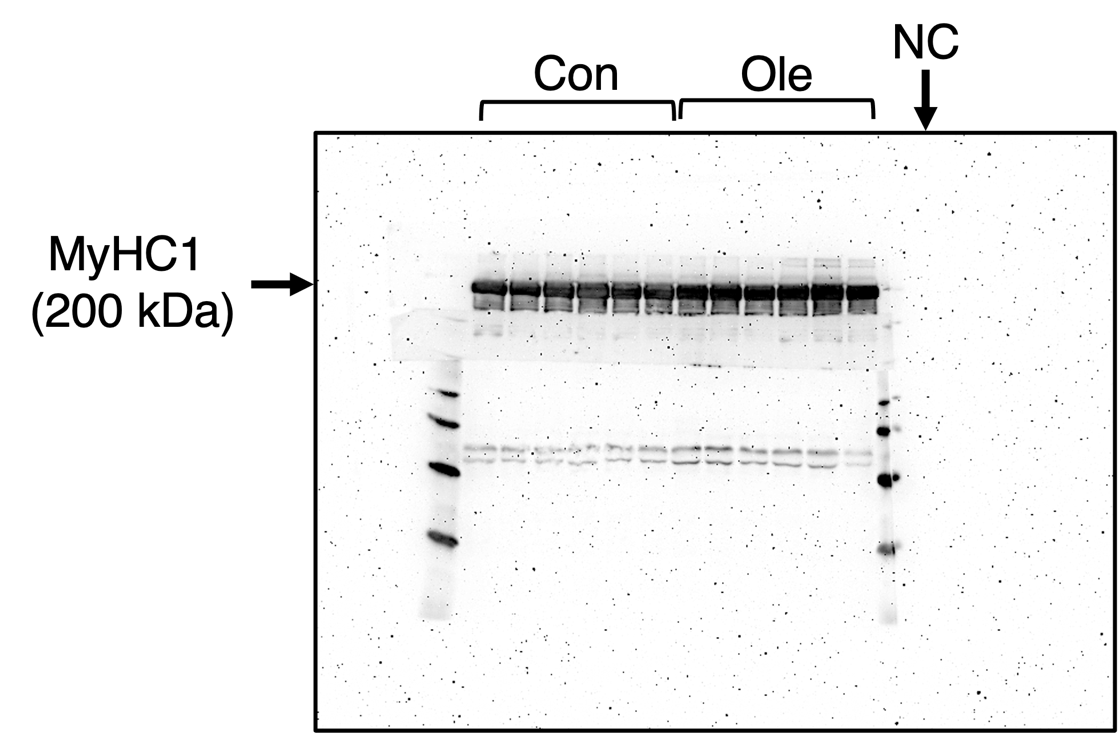
**

**＜Fig. 2C Soleus MyHC2＞**

**
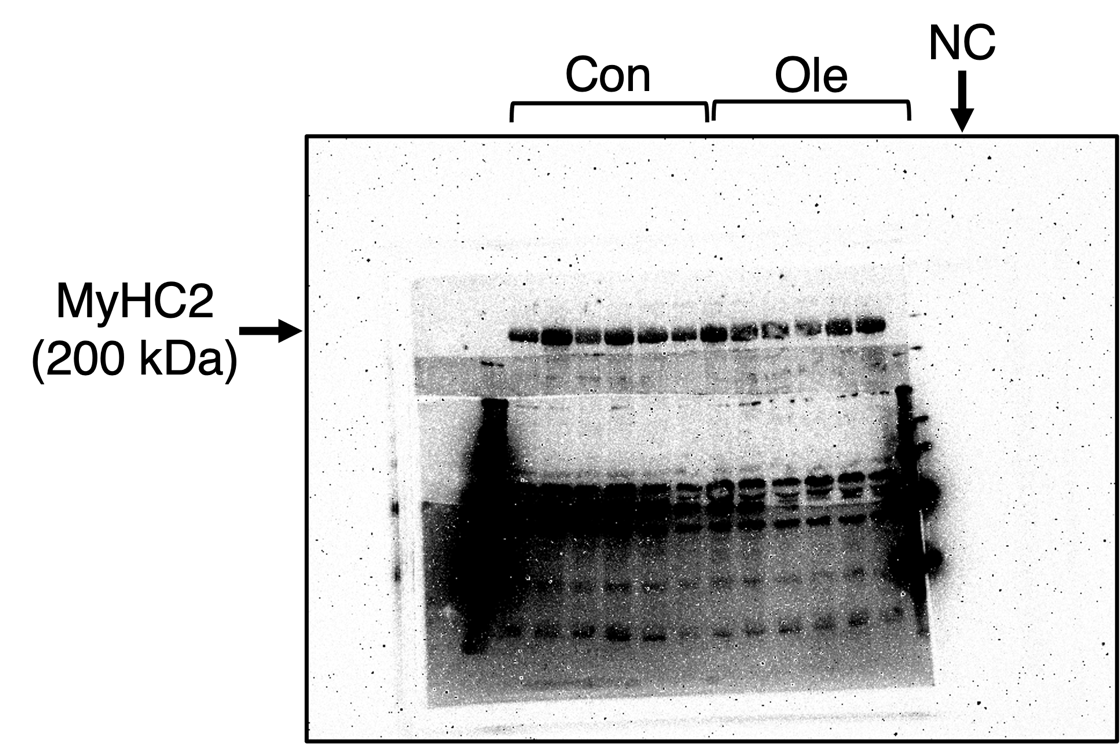
**

**＜Fig. 2C Soleus PGC-1α＞
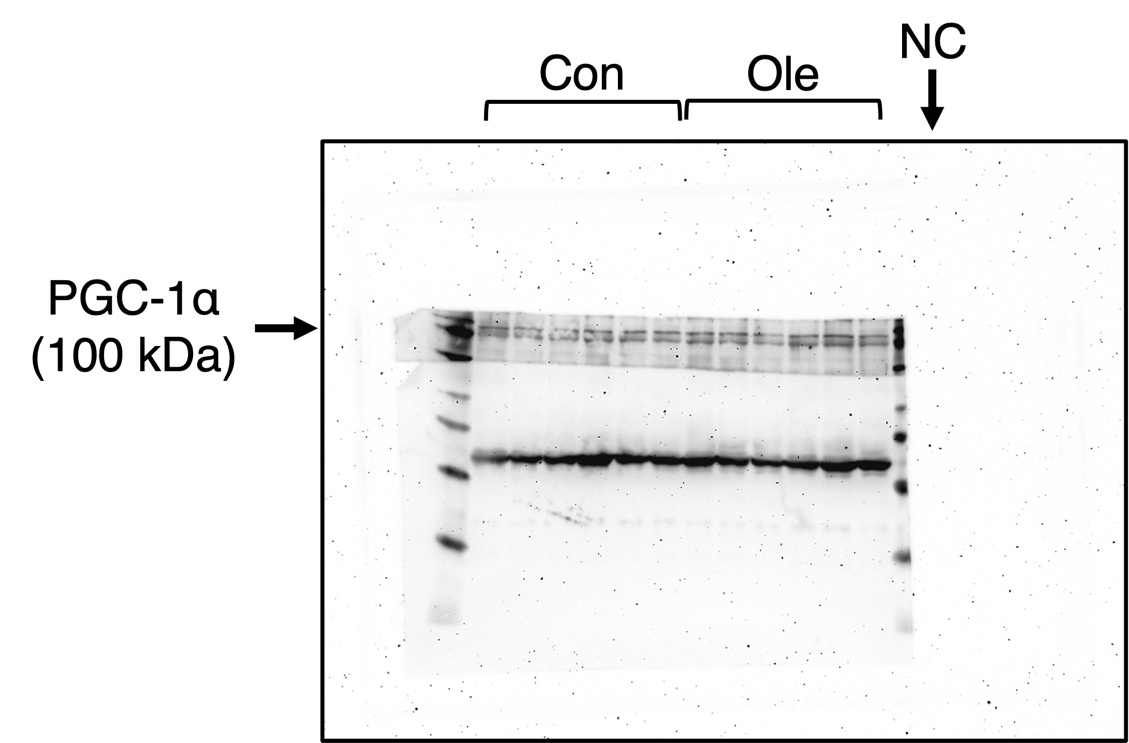
**

30

50

40

60

20

80

100

120

**＜Fig. 2C Soleus PGC-1β＞**

**
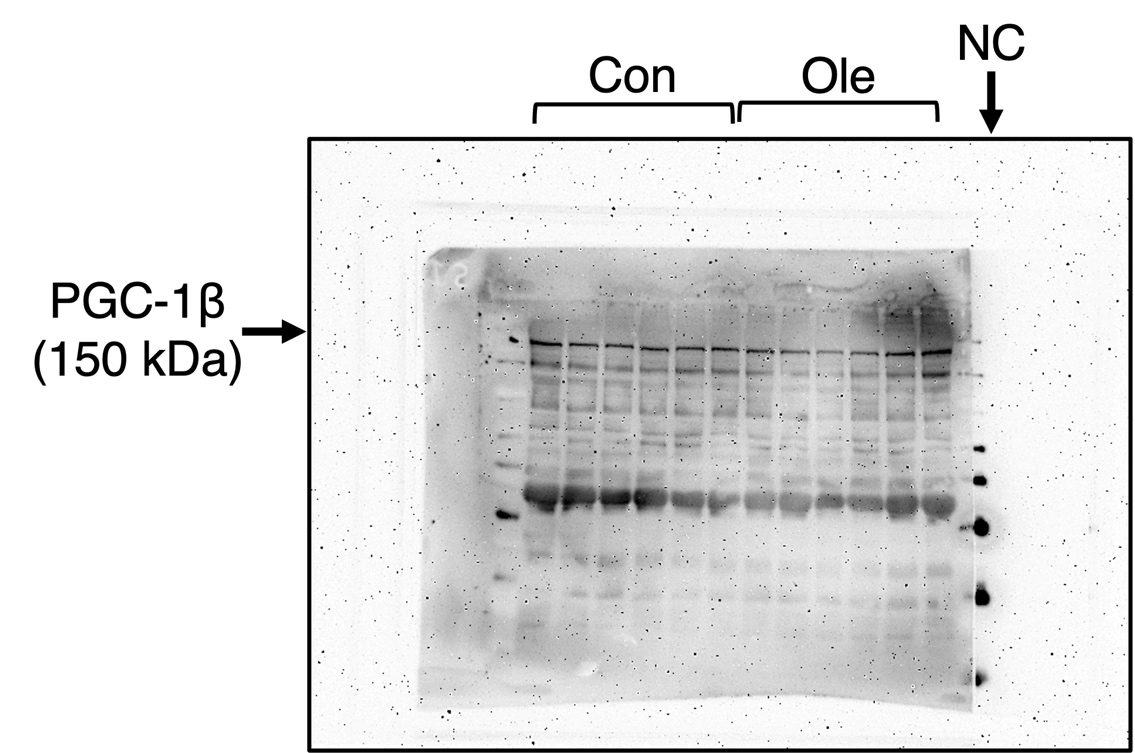
**

**＜Fig. 2C Soleus Actin＞**

**
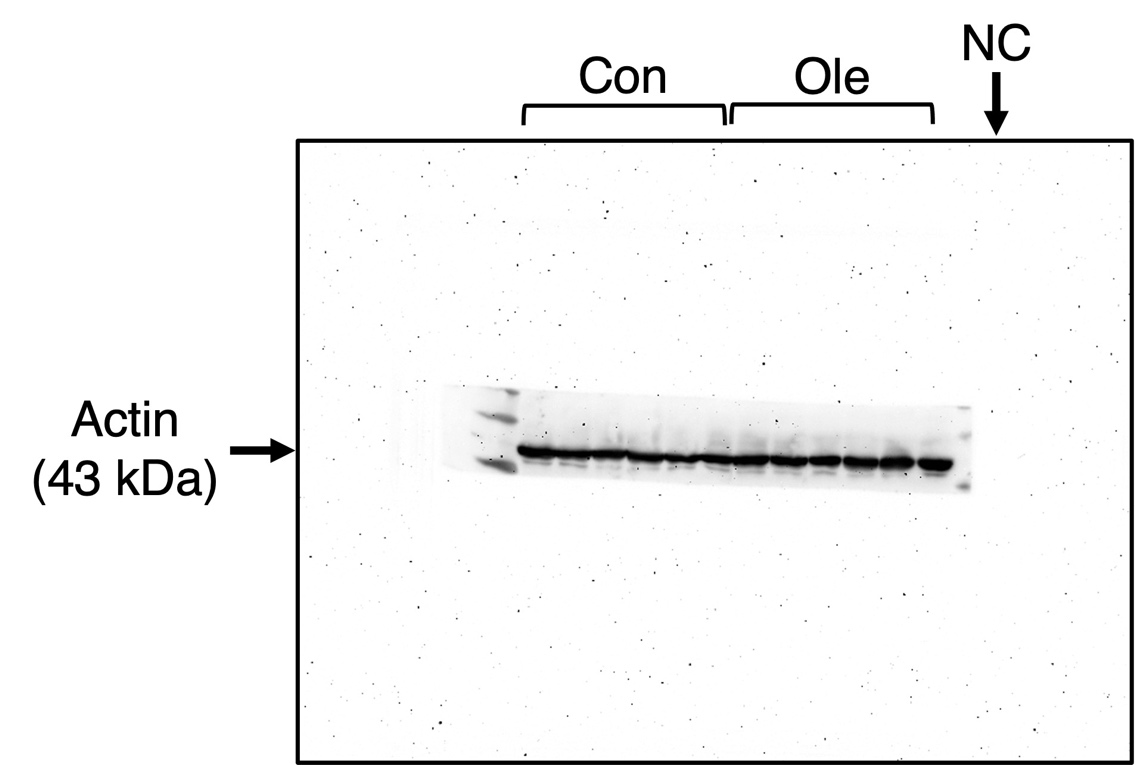
**

**＜Fig. 2C EDL MyHC1＞**

**
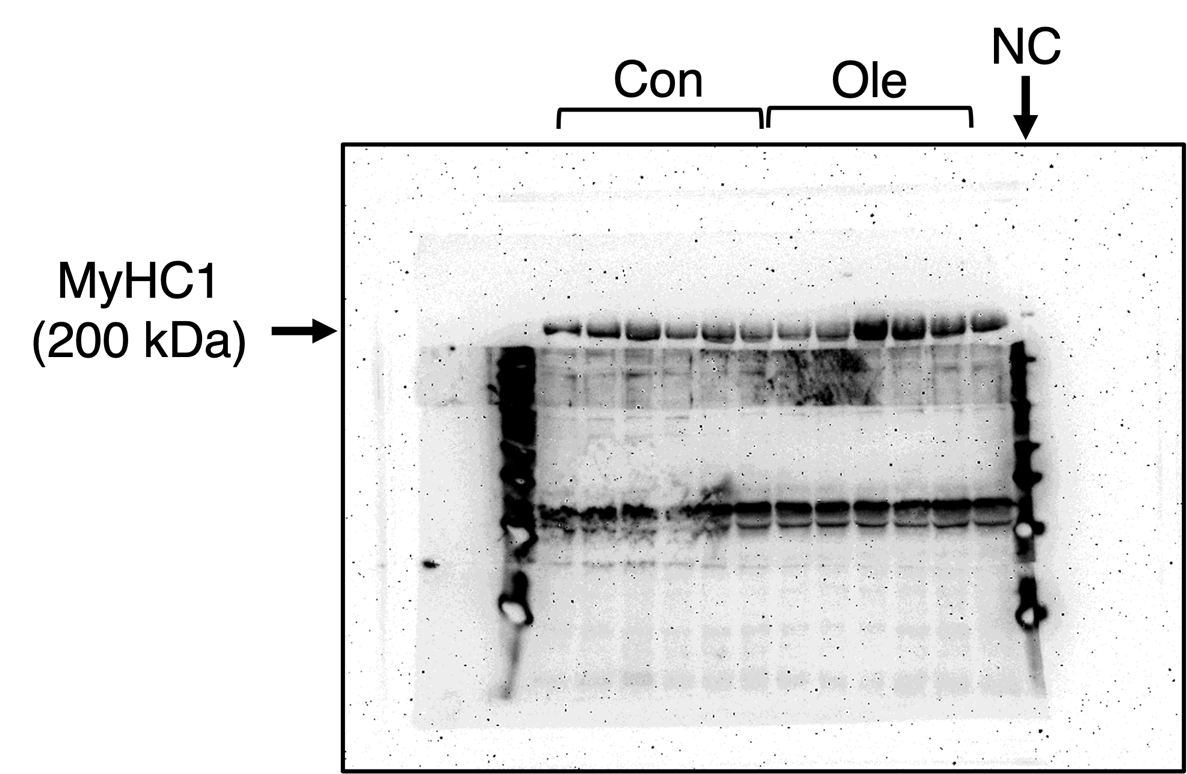
**

**＜Fig. 2C EDL MyHC2＞**

**
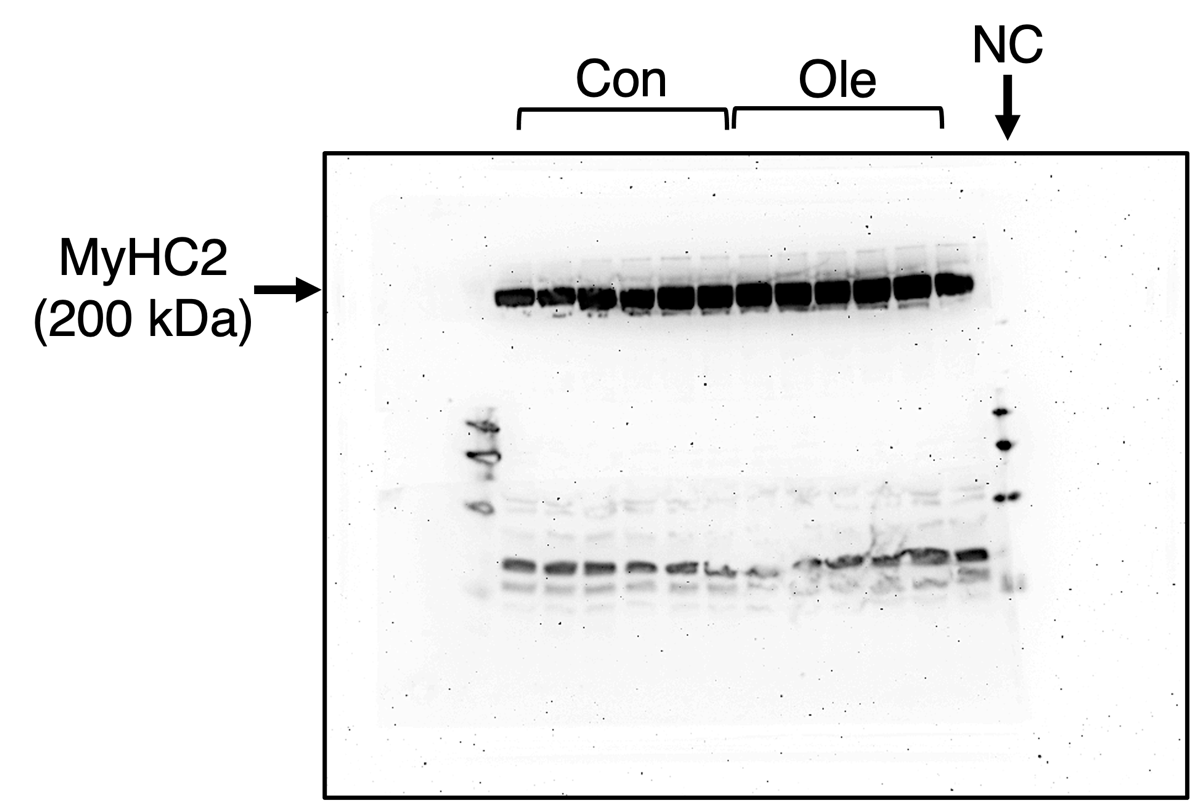
**

**＜Fig. 2C EDL PGC-1β＞**

**
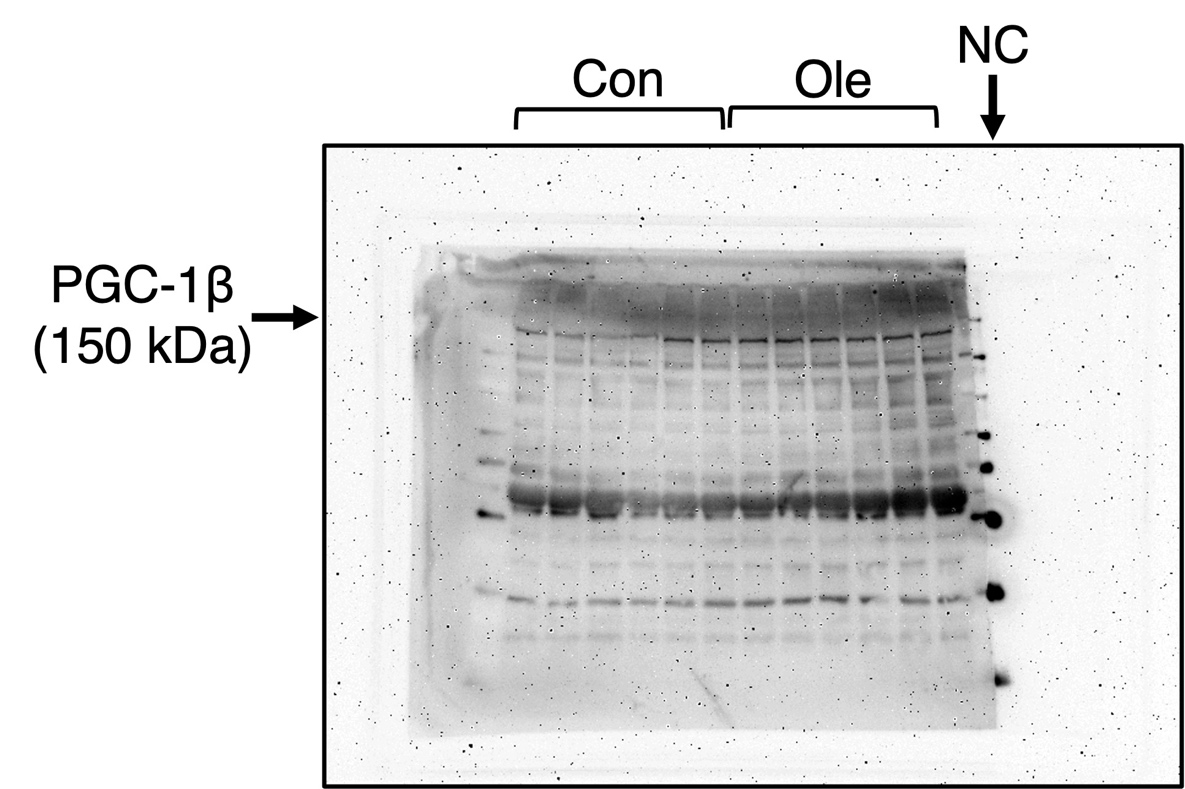
**

**＜Fig. 2C EDL Actin＞**

**
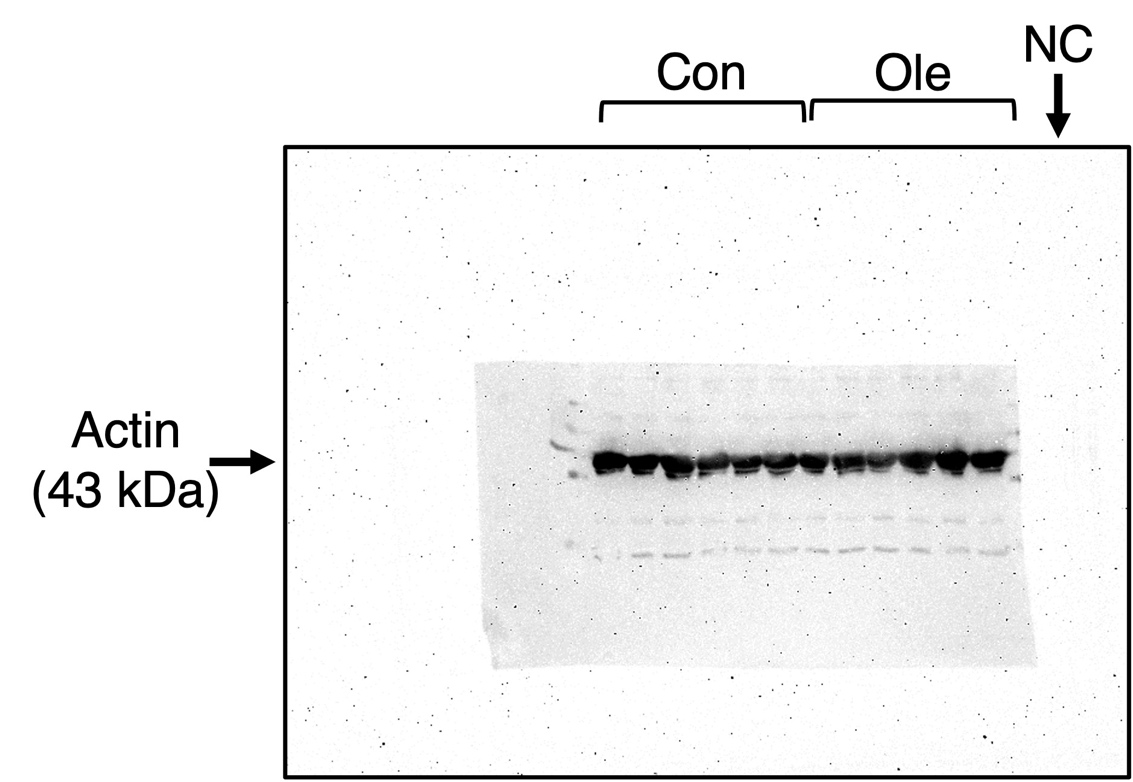
**

**＜Fig. 4A Soleus CPT1B, Porin＞**

**
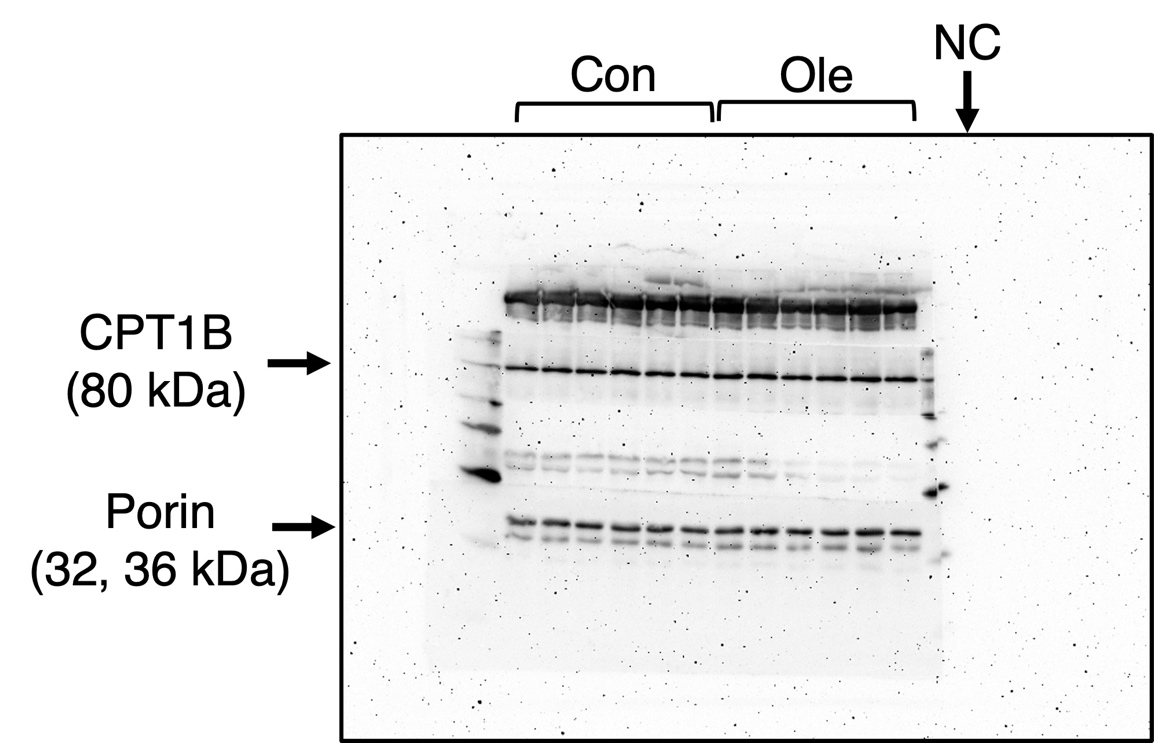
**

**＜Fig. 4A Soleus PDK4＞**

**
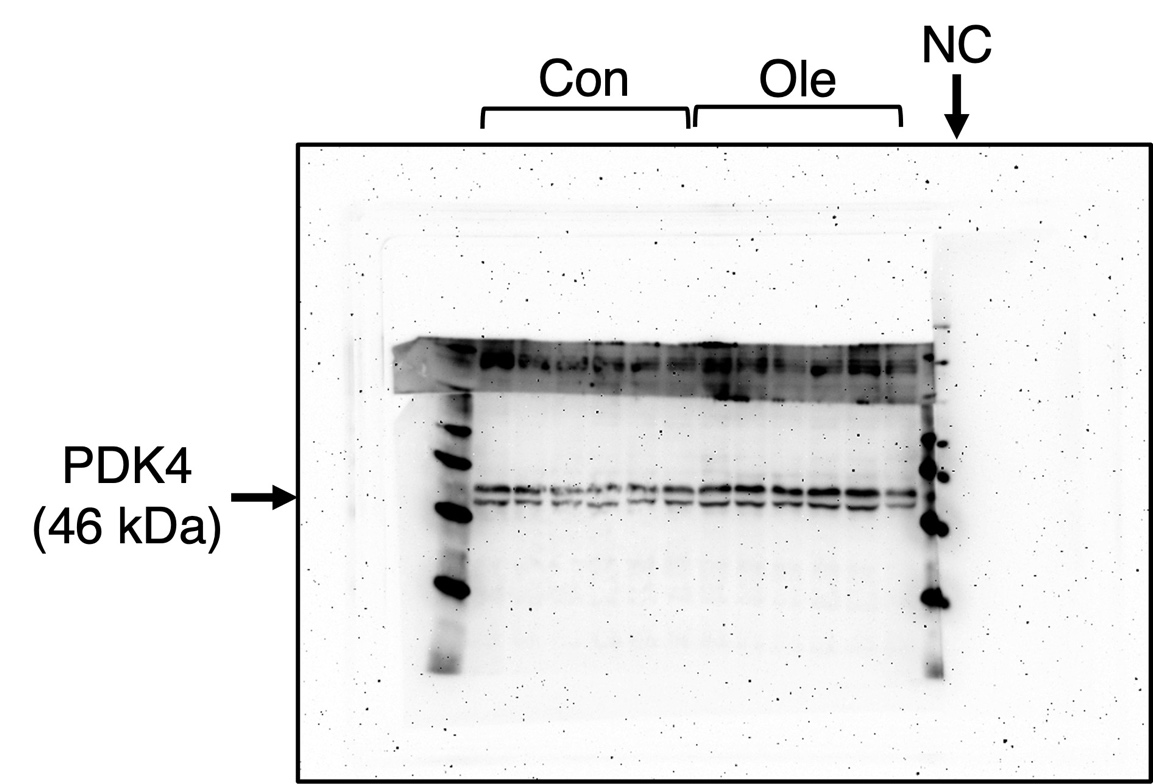
**

120

100

80

60

20

30

40

50

**＜Fig. 4A Soleus Actin＞**

**
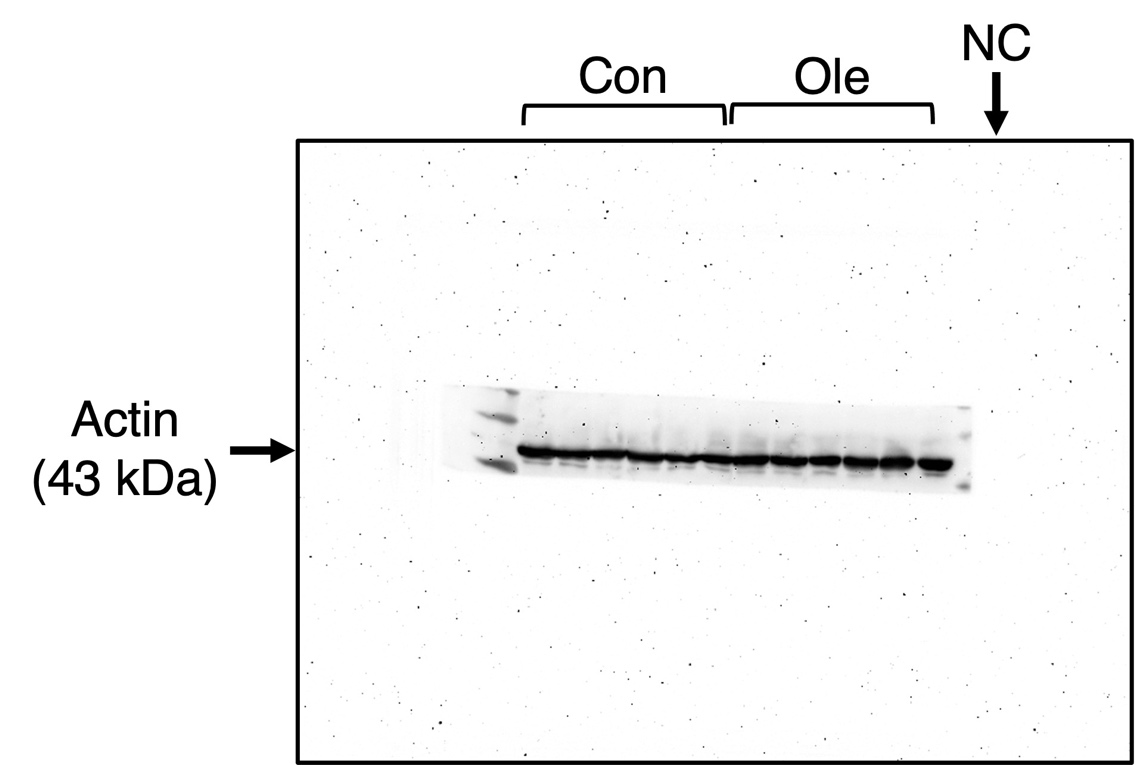
**

**＜Fig. 4D EDL CPT1B, Porin＞**

**
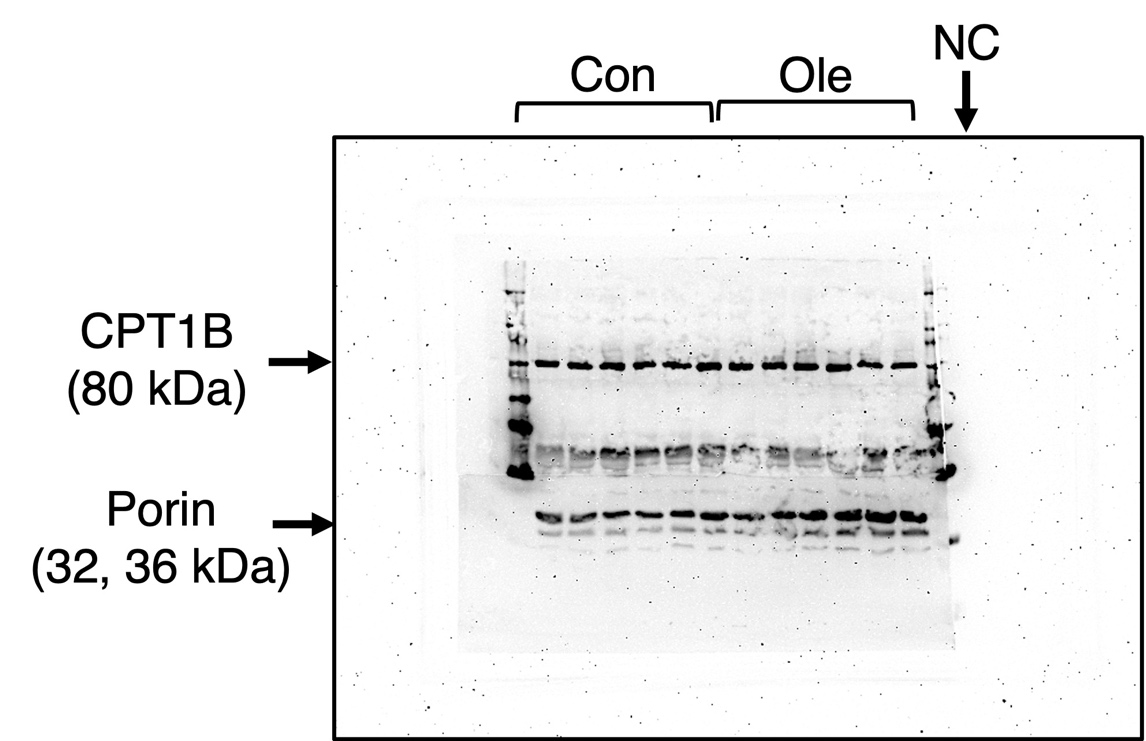
**

**＜Fig. 4D EDL Actin＞**

**
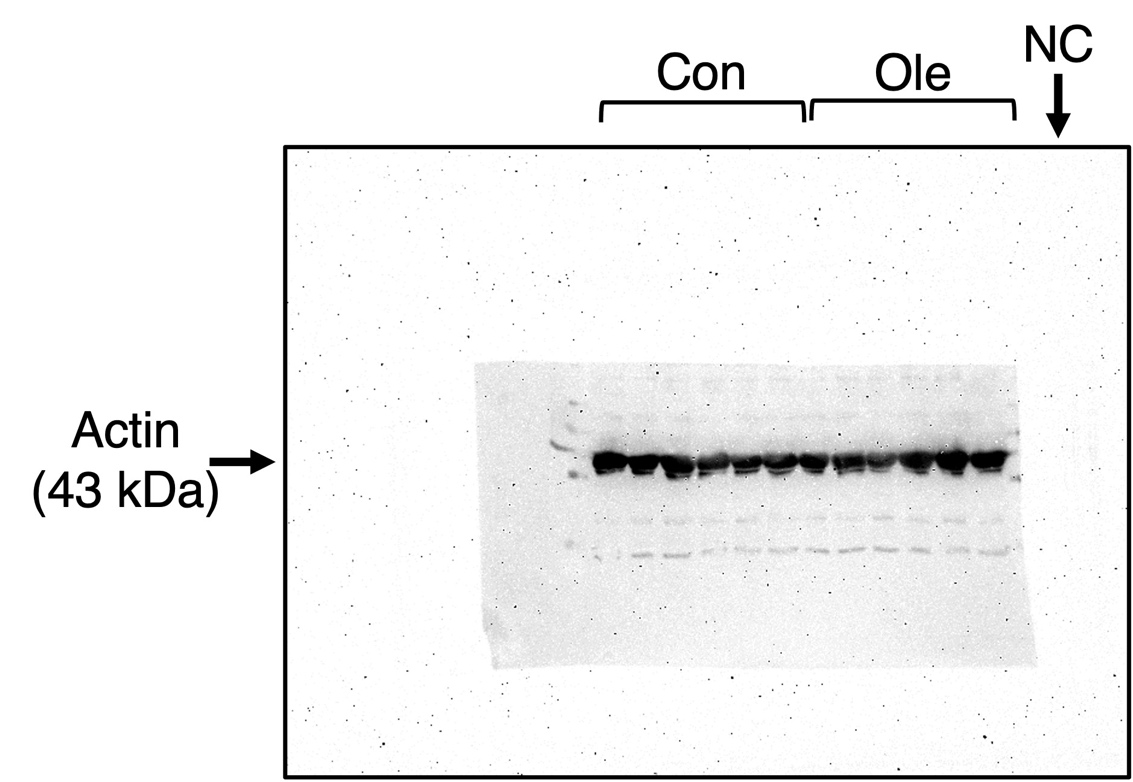
**
